# How Does Information Sharing of a Supervisor Influence Proactive Change Behavior of an Employee? The Chain Mediating Role of Family-Like Employee–Organization Relationship and Relationship Energy

**DOI:** 10.3389/fpsyg.2021.739968

**Published:** 2021-12-23

**Authors:** Xiao-Xia Zhu, Chun Li, Xiao-Ling Wang, Jun-Na Liu, Senmao Xia

**Affiliations:** ^1^School of Management, Shanghai University, Shanghai, China; ^2^Department of Human Resource Management, Shanghai Normal University, Shanghai, China; ^3^School of International Relations and Public Affairs, Fudan University, Shanghai, China; ^4^International Center for Transformational Entrepreneurship, Coventry University, Coventry, United Kingdom

**Keywords:** work-related information sharing of supervisors, non-work information sharing of supervisors, family-like employee–organization relationship, relationship energy, proactive change behavior

## Abstract

The proactive change behavior of an employee is the key to promoting organizational innovation. However, the proactive change has a certain risk, and many employees are unwilling to implement initiatively. How to promote the occurrence of a proactive change behavior of an employee has become a hot issue in the theoretical and practical areas. Based on the self-disclosure theory, this study uses the questionnaire survey method, containing a total of 32 items, and uses the 5-point Likert scale (1 = strongly disagree and 5 = strongly agree), with the Mplus and SPSS statistical software to analyze the impact mechanism of work-related information sharing of supervisors on the proactive change behavior of employees through the structural equation model. The regulatory effect of non-work information sharing of leaders is analyzed using the latent regulatory structural equation method. The conclusions are as follows: work-related information sharing positively of supervisors influences the family-like employee–organization relationship of employees; the family-like employee–organization relationship and relationship energy play serial mediating roles in the relationship between work-related information sharing of supervisors and the proactive change behavior of employees; non-work information sharing of supervisors moderates the serial mediating path by enhancing the positive influence of work-related information sharing of supervisors on the family-like employee–organization relationship. Theoretically, this study has complemented and enriched the research on the influence mechanism between the information sharing of supervisors and the proactive change behavior of employees. Practically, this study has important implications for supervisors to promote the proactive change behavior of employees by sharing work-related information and non-work information with employees.

## Introduction

In VUCA era, the economic environment of enterprises is shrouded by great uncertainty. Only by continuous innovation can enterprises improve their adaptability. In enterprise practice, the proactive change behavior of an employee is an important way to improve the organizational innovation ability (Kim and Liu, 2017). Previous studies have shown that the proactive change behavior of an employee has a positive effect on improving the working engagement, working performance, organizational adaptability, and innovation of employees (Müceldili and Erdil, 2016; Wu et al., 2017). However, due to the inherent challenges and risks of the proactive change behavior, and the emphasis on “favor” and “face” in the Chinese context, some employees only focus on tasks within their roles to avoid resource losses and interpersonal conflicts (Zhang et al., 2020). Therefore, how to motivate employees to “be willing” to implement the proactive change behavior has gained a great interest from academia and management practitioners.

The proactive change behavior of employees refers to their voluntary and constructive efforts to achieve the transformation of organizational functions (Morrison and Phelps, 1999). Existing studies have shown that transformational leadership, participatory leadership, and empowering leadership can promote the proactive change behavior of employees (Zhang et al., 2018; Hao and Long, 2020; Wang et al., 2020). However, few studies focus on the impact of information sharing behavior of supervisors on the proactive change behavior of employees. Some scholars pointed out that the task of supervisors is not only to establish and maintain relationships with employees but also to transfer information (Xu and Ouyang, 2012). Work-related information sharing of supervisors can cultivate overall consciousness and responsibility cognition of employees through information transmission. It can not only positively affect the employee performance but also promote working engagement of employees (Chen et al., 2018). So, will the information sharing behavior of supervisors also encourage employees to take proactive change behavior? This issue needs urgent attention.

Based on the “Guanxi,” this study identified two specific variables that may have an impact on work-related information sharing of supervisors and the proactive change behavior of employees, including the family-like employee–organization relationship and relationship energy. The family-like employee–organization relationship means that under the background of high collectivism and “family culture,” employees and their enterprises meet the needs of each other, regardless of gains and losses, and share weal and woe (Zhu et al., 2015). Relationship energy is a useful psychological resource produced by a positive relationship, which can effectively deal with the pressure and burnout in the workplace, so as to improve the work ability of employees. In the process of studying how the behavior of supervisors affects the proactive change behavior of employees, their own feelings and emotions are important mediating factors (Chen et al., 2006; Chen and Aryee, 2007). In addition, in the context of Chinese culture, “Guanxi” plays an important role in the formation of feelings and emotions of employees. According to the self-disclosure theory, disclosure of information to others can effectively predict relationship satisfaction and relationship quality. Work-related information sharing of supervisors to some extent reflects their trust in employees, helps to improve their internal identity recognition, and promotes them to establish a close emotional link with the organization, resulting in a family-like employee–organization relationship. The relationship energy generated by employees in this positive relationship can provide psychological resources for employees to creatively solve organizational problems and implement the proactive change behaviors. To sum up, this study will establish a chain-mediated model of work-related information sharing of supervisors influencing the proactive change behavior of employees through the family-like employee–organization relationship and relationship energy.

The data show that leaders spend 70% of their daily time communicating with employees (Jablin, 1979). Effective communication between them can create a good working atmosphere (Rasool et al., 2020). Besides work-related information, their communication contents may also involve hobbies, spare time of life, and non-work information sharing related to family. According to the self-disclosure theory, the disclosure of personal information to others is conducive to developing positive relationships and thus affecting the regulation of individual behavior further (Jiang et al., 2008). Non-work information sharing of supervisors can reduce the power distance between employees and supervisors, close the relationship between employees and organizations, and thus deeply affect the behavior of employees. Therefore, this study intends to explore how the non-work information sharing of supervisors moderates the chain-mediated effect of the family-like employee–organization relationship and relationship energy between the work-related information sharing of supervisors and the proactive change behavior of employees.

**FIGURE 1:**
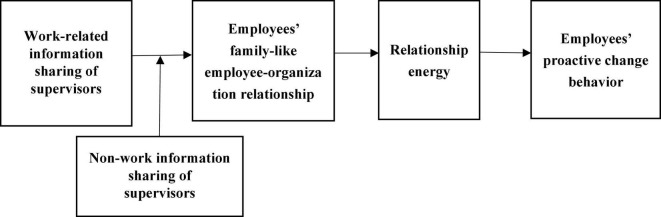
Hypothesized model.

The article is structured as follows: the second part presents the relevant research on information sharing of supervisors, family-like employee–organization relationship, relationship energy, and proactive change behavior of employees, makes research hypotheses, and constructs the theoretical model as shown in Figure 1. The third part is the methods. The fourth part is the data analysis. The last part is the research conclusion and discussion.

## Theory and Hypothesis

### Work-Related Information Sharing of Supervisors and the Family-Like Employee–Organization Relationship of Employees

The family-like employee–organization relationship shows the family-like cooperative relationship between employees and the organization. As a representative of the enterprise, the behavior of supervisors is an important contextual factor, which affects the relationship between employees and the organization, and work-related information sharing of supervisors is the most common behavior of supervisors. Work-related information sharing of supervisors refers to their dissemination of mission, ideas, and other information within the company, including explaining to employees the decision-making and the development strategies of the company (Nifadkar et al., 2019). According to the self-disclosure theory, sharing information with interactive partners can promote both parties to develop a positive relationship (Jones and Archer, 1976; Archer and Cook, 1986). Willingness of supervisors to share more work-related information with employees conveys their trust in employees, which reflects the high-quality relationship between supervisors and employees. Employees with high-quality leadership exchange relationships generally have stronger psychological contracts with the organization (Hogan and Holland, 2003) and are more likely to have the family-like employee–organization relationship. Information is the foundation of power of supervisors (Amundsen and Martinsen, 2014). Sharing of work-related information by supervisors to employees means that they share power with employees, so that “insiders” identity and organizational status of employees can be recognized, which is conducive to enhancing the willingness of employees to serve the organization for a long time (Song et al., 2009; He et al., 2020) and promoting the formation of the family-like employee–organization relationship of employees in the process of organizational integration. Work-related information sharing of supervisors enables employees to understand the mission and goals of the company, as well as the role and meaning of their positions, which helps employees view their contributions to achieve organizational goals from the corporate level and enhances their sense of work meaning (Chen et al., 2017). It is also conducive to promoting employees from the “little self” to the “greater self,” thereby forming a blended relationship of “from the little self to the greater self.”

Existing research shows that work-related information sharing of supervisors can positively affect the relationship between employees and the organization. Scholars such as Chen et al. (2017) pointed out that work-related information sharing of supervisors reflects their affirmation of abilities of employees, which can strengthen their internal cognition. In addition, enough information is the basis for effective decision-making. Work-related information sharing of supervisors can increase the sense of control of employees over their work, positively affect their work engagement, and establish a close emotional link with their work (Chen et al., 2018). Therefore, this article speculates that work-related information sharing of supervisors can positively affect the family-like employee–organization relationships of employees.

Based on the above discussion, the following research hypothesis is proposed:

H1: Work-related information sharing of supervisors has a positive impact on family-like employee–organization relationships of employees.

### Family-Like Employee–Organization Relationship and Relationship Energy

Relationship energy reflects an improved level of psychological resources of employees in a relationship, which is conducive to enhancing their work ability (Owens et al., 2016). The family-like employee–organization relationship is the display of the positive relationship between employees and the organization. This positive relationship can provide employees with deep-seated psychological resources, which is vital to the generation of relationship energy. When there is a kind of the family-like employee–organization relationship, employees and the organization realize the deep emotional embedding of “You have me, and I have you.” It can bring happiness and satisfaction to employees for working in the organization, thus put employees in a dynamic emotional state and have more relationship energy. The family-like employee–organization relationship embodies pan-family feeling of mutual dependence and integration between employees and the organization essentially, which makes it easier to perceive psychological resources such as self-identity and social attachment in the process of interacting with organization members, thereby generating relationship energy. According to the altruistic rule followed by the family-like employee–organization relationship, when employees and organizations are in this relationship, employees will treat supervisors and organizations as relatives (Wang et al., 2018), which can inspire employees to be willing to make effort. Moreover, in this process, they would gain more physical, emotional, and psychological resources and have more relationship energy. In contrast, employees who have no family-like employee–organization relationship will maintain clear boundaries with the organization and put greater emphasis on the equality of rights and obligations and the ratio of input and return in its work. Moreover, their emotional commitment to the organization is relatively low (Zhu et al., 2015), their work motivation is shortage, and their relationship energy is less.

An empirical study by Wang et al. (2018) pointed out that the family-like employee–organization relationship can positively affect assistance behaviors of employees. The reason is that the family-like employee–organization relationship is relatively stable compared with other relationships. It can stimulate positive attitudes of employees and may become an internal incentive of employees, that is, relationship energy. Zhu et al. (2015) showed that the family-like employee–organization relationship can enhance positive behaviors of employees such as emotional commitment and organizational citizenship. This reveals that the family-like employee–organization relationship may stimulate positive energy of employees, that is, relationship energy.

Based on the above discussion, the following research hypothesis is proposed:

H2: Family-like employee–organization relationship has a positive impact on relationship energy.

### Mediation Effect of the Family-Like Employee–Organization Relationship in Work-Related Information Sharing of Supervisors Relationship Energy

Work-related information sharing of supervisors can provide employees with the work support they need, reduce uncertainty and risk concerns in working, help avoid possible differences and conflicts between supervisors and employees, and build a harmonious atmosphere similar to getting along with relatives, which promotes the formation of family-like employee–organization relationships (Long and Chen, 2020). This kind of relationship can improve effort-achievement expectations and goal-achievement intrinsic valence of employees (Wang et al., 2009; Tan et al., 2019) and provide motivation for their hard work, so that relationship energy is generated. Work-related information sharing of supervisors indicates that supervisors recognize this employment contract relationship and appreciate the personal value of employees, which is conducive to shortening the distance between the superior and the subordinate, increasing mutual emotional communication and promoting the formation of family-like employee–organization relationships. By providing employees with social and emotional resources, supervisors can stimulate their inner work motivation and generate relational energy. In addition, work-related information sharing of supervisors indicates that there may be a close psychological contract connection between the leader and the employee. This connection will affect the self-positioning and cognition of employees, promoting the formation of the family-like employee–organization relationship, and then affect their psychological resources to complete tasks (Sonnentag et al., 2012). Therefore, we believed that this “strong relationship” with the supervisor can promote the formation of the family-like employee–organization relationship and then, generate relationship energy.

The studies have shown that management behavior can affect psychological resources of employees by changing their subjective cognition (He et al., 2021). Scholars such as Wu et al. (2017) pointed out that the practice of benevolence-oriented human resources can positively influence the in-role behavior and out-of-role behavior of employees through the family-like employee–organization relationship. It is easy to see that the benevolent management treating employees as relatives can stimulate role recognition of employees as relatives and provide psychological resources and relationship energy for employees in implementing positive behaviors. In addition, scholars such as Wang et al. (2019) have proved that the organizational relationship of employees can positively affect emotional commitment of employees through the family-like employee–organization relationship. The investment behavior of an organization in employees is conducive to changing perceptions of their own responsibilities of employees, making them more persevering in working. Thus, we assumed:

H3: Work-related information sharing of supervisors can positively affect relationship energy through the family-like employee–organization relationship.

### The Serial Mediation Effect of the Family-Like Employee–Organization Relationship and Relationship Energy

The proactive change behavior has certain risks due to the uncertainty of its results. Whether employees taking change largely depends on the evaluation of changing outcome and their own work level (Zhang et al., 2018). The relationship energy generated during the interaction between supervisors and employees can enhance psychological resources and work capabilities of employees, which is a key factor affecting the proactive change behavior of employees. Relationship energy is also a resource transfer mechanism. The relationship energy that employees obtain from the workplace is likely to be transformed into proactive change behaviors that can inject energy into the organization (Owens et al., 2016). According to the principle of reciprocity, when employees obtain relationship energy from their supervisors, they will reward the organization with loyalty and extra effort, take responsibility initiatively, and then take change. Therefore, relationship energy plays an important role in the generation of the proactive change behavior of employees. In addition, research by Onyishi and Ogbodo (2012) found that an important feature of relationship energy is that the improvement of self-efficacy has a significant positive correlation with the proactive change behavior of employees.

Based on the above discussion, we proposed the following hypothesis:

H4: Relationship energy can positively affect the proactive change behavior of employees.

Combining Hypothesis 3 and Hypothesis 4, this study holds that work-related information sharing of supervisors can further promote the generation of the proactive change behavior of employees through the mediating transmission of relationship energy. Specifically, work-related information sharing of supervisors is an embodiment of organizational support, which enables employees to feel the trust and recognition of their abilities and values from the leaders and the organizations, which is conducive to the formation of the family-like employee–organization relationship among employees. Once such a positive relationship with the organization is formed, employees are more likely to feel emotional and psychological resources from this relationship and trigger their positive emotions, including relationship energy. This contributes to enhancing the psychological function of employees in work, so that employees are more willing to invest more time and energy for the success of the organization, shoulder more responsibilities, and take the proactive change behavior.

To sum up, we assumed that:

H5: Family-like employee–organization relationship and relationship energy play serial mediating roles in the relationship between work-related information sharing of supervisors and the proactive change behavior of employees.

### The Moderation Effect of Non-work Information Sharing of Supervisors

In the process of the interaction between superiors and subordinates, in addition to work-related information sharing, supervisors often share non-work information with employees. Non-work information sharing refers to the communication between superiors and subordinates about their concerns, interests, and activities outside the organization (Nifadkar et al., 2019).

According to the self-disclosure theory, a high level of non-work information sharing of supervisors may mean that employees are intimate to supervisors and have a closer psychological distance with supervisors, so that they can be trusted by supervisors (Nifadkar et al., 2019). Emotional trust is more likely to make the relationship between superiors and subordinates developed in a harmonious direction (Shen et al., 2019). In the context of harmonious organizational atmosphere and high power distance culture, employees tend to regard leaders as the embodiment of the organization. When superiors share work information, employees are more likely to internalize the mission and philosophy of the organization and are more willing to devote themselves to the affairs of the company, which is conducive to the formation of the family-like employee–organization relationship. In another word, when the degree of non-work information sharing of supervisors is high, their work-related information sharing has a stronger positive impact on the family-like employee–organization relationship. On the contrary, when the degree of non-work information sharing of supervisors is low, the intersection of supervisors and employees involves almost exclusively work. For Chinese people who emphasize human feelings and relationships, there is still a certain distance to harmonious relationship atmosphere. Employees may interpret work-related information sharing of supervisors as an expectation that they will reciprocate at work. This kind of assistant behavior will not be regarded as unconditional contribution by employees and may reduce the willingness of devoting themselves to the organization. Therefore, when the degree of non-work information sharing of supervisors is low, their work-related information sharing has a weaker impact on the family-like employee–organization relationship.

At the same time, non-work information sharing of supervisors will strengthen the positive impact of work-related information sharing of supervisors on the family-like employee–organization relationship, so as to have a moderated effect on the serial-mediated effect of the family-like employee–organization relationship and relationship energy. Specifically, when the degree of non-work information sharing of supervisors is high, it is conducive to promoting the establishment of workplace friendship between supervisors and employees. In these circumstances, employees may be inclined to regard their supervisors as relatives and friends (Liu et al., 2020). When supervisors are sharing work-related information, emotional Chinese employees would not feel that their relationship with supervisors and organization is merely instrumental and may think that supervisors can not only provide emotional support but also take care about their careers. It is more likely to make employees feel the family-like employee–organization relationship and experience more relationship energy, so as to implement the proactive change behavior.

To sum up, we proposed the following research hypothesis:

H6: By strengthening the positive effect of work-related information sharing of supervisors on family-like employee–organization relationship, their non-work information sharing moderates the serial-mediated effect of family-like employee–organization relationship and relationship energy on work-related information sharing of supervisors and the proactive change behavior of employees.

## Methods

### Research Approach

This research uses the questionnaire survey method to collect the data. The reasons for adopting this method are mainly based on the following points. First, the behavior of supervisors will directly affect employees, so it is reasonable to measure this impact through self-report of employees (Zhou et al., 2021). Second, the questionnaire is anonymous, and the respondents can express their true wishes. Finally, a large number of valid questionnaires collected can test the scientific characteristics of the research hypothesis.

### Research Samples

The questionnaire respondents came from Shanghai, Sichuan, Beijing, Guangdong, Heilongjiang, and other provinces and cities, involving enterprises with different natures such as state-owned enterprises, foreign enterprises, and private enterprises. We sent out 600 questionnaires totally and collected 455 questionnaires finally through the online platform. The response rate is 75.8%. To ensure the validity of the analysis, we eliminated 34 invalid questionnaires. The effective rate is 70.2%.

According to the descriptive statistics of samples, males and female account for 46.1 and 53.9%, respectively; most of them are aged from 21 to 30 years old (66.3%) and have bachelor’s degree (49.4%); most of the employees work in private enterprises (29.2%); and most of them have worked for less than 3 years (69.3%). The position level is concentrated in ordinary employees (84.6%), and the company size is less than 100 employees (33.7%).

### Measure Instruments

We used the six-item scale developed by Nifadkar et al. (2019) to measure the work-related information sharing of supervisors and their non-work information sharing, items including, “my boss will let me know the reason why my job task changes,” “my boss will tell me some important changes in his/her home” (1 = strongly disagree and 5 = strongly agree), and so on. Cronbach’s α values were 0.859 and 0.955 (>0.7), respectively.

We used five-item scale developed by Owens et al. (2016) to measure relationship energy, items including “when I interact with my boss, I feel energized” (1 = strongly disagree and 5 = strongly agree) and so on. Cronbach’s α was 0.871.

We used five-item scale developed by Zhu et al. (2015) to measure the family-like employee–organization relationship, items including, “I put the interests of the company first in work, because the interests of the company are closely related to my interests” (1 = strongly disagree and 5 = strongly agree) and so on. Cronbach’s α was 0.879.

We used the 10-item scale developed by Morrison and Phelps (1999) to measure the proactive change behaviors of employees, items including “I usually try to adopt a better way to complete my job at work” (1 = strongly disagree and 5 = strongly agree) and so on. Cronbach’s α was 0.933.

Previous studies have shown that background variables such as gender, age, education background, working years, and position level of employees, and nature and size of the company have impact on their proactive change behavior (Hao and Long, 2020). Therefore, the above variables are used as control variables in this study.

## Data Analysis

### Validity Testing

In this study, the confirmatory factor analysis was used to verify the discriminant validity among the five variables by Mplus, namely, work-related information sharing of supervisors, their non-work information sharing, relationship energy, proactive change behavior, and family-like employee–organization relationship.

The results are shown in Table 1. Among all the models, the five-factor model has goodness of fit index.

**TABLE 1:** Fitting index of the scale by the confirmatory factor analysis.

Model	χ^2^	df	χ^2^/df	RMSEA	CFI	TLI	SRMR
Five-factor model IS, NIS, RE, LQC, PC	965.766	454	2.127	0.052	0.942	0.937	0.055
Four-factor model IS + NIS, RE, LQC, PC	1434.077	458	3.131	0.071	0.890	0.881	0.070
Three-factor model IS + NIS + RE, LQC, PC	2204.604	461	4.782	0.095	0.803	0.788	0.096
Two-factor model IS + NIS + RE + LQC, PC	3069.342	463	6.629	0.116	0.706	0.685	0.115
Single-factor model IS + NIS + RE + LQC + PC	5483.875	464	11.819	0.160	0.433	0.394	0.190

*N = 421.*

*CFI, comparative fit index; IS, work-related information sharing of supervisors; NIS, non-work information sharing of supervisors; RE, relationship energy; RMSEA, root mean square error of approximation; SRMR, standardized root mean square residual; TLI, Tucker–Lewis index; LQC, family-like employee–organization relationship; PC, proactive change behavior.*

### Descriptive Statistics Analysis

In this part, we mainly carried out the descriptive statistics and correlation statistics of each variable, and the mean, standard deviation, and correlation coefficient are shown in Table 2. In Table 2, work-related information sharing of supervisors (*r* = 0.148, *P* < 0.01), their non-work information sharing (*r* = 0.155, *P* < 0.01), and relationship energy (*r* = 0.219, *P* < 0.01) are all positively correlated with the proactive change behavior of employees. Work-related information sharing of supervisors (*r* = 0.278, *P* < 0.01) and their non-work information sharing (*r* = 0.408, *P* < 0.01) are positively correlated with relationship energy. These results provide a preliminary basis for some hypotheses proposed in this study. At the same time, it can be seen in Table 2 that the synchronous changes among variables are of statistical significance, which can be used for the regression analysis.

**TABLE 2:** Correlation coefficient and descriptive statistics of variables.

	Mean	SD	1	2	3	4	5	6	7	8	9	10	11	12
1. IS	3.91	0.712	1											
2. NIS	3.27	1.170	0.594**	1										
3. RE	3.73	0.784	0.278**	0.408**	1									
4. LQC	3.93	0.723	0.353**	0.337**	0.292**	1								
5. PC	3.77	0.786	0.148**	0.155**	0.219**	0.177**	1							
6. Gender	–	–	–0.020	–0.026	–0.085	0.038	0.009	1						
7. Age	–	–	0.106*	0.303**	0.215**	0.145**	0.103*	–0.043	1					
8. Education background	–	–	−0.148**	−0.326**	−0.131**	−0.117*	−0.160**	0.036	−0.299**	1				
9. Working years	–	–	0.126**	0.327**	0.185**	0.130**	0.189**	−0.161**	0.280**	−0.341**	1			
10. Position level	–	–	–0.003	0.054	0.030	–0.038	–0.010	–0.009	0.120*	–0.085	0.105*	1		
11. Nature of company	–	–	0.000	−0.173**	−0.107*	–0.050	−0.107*	0.073	−0.128**	0.022	–0.087	–0.057	1	
12. Size of company	–	–	0.140**	0.133**	0.082	0.103*	0.001	–0.069	0.014	–0.037	0.036	0.001	−0.162**	1

*N = 421.*

**Means that correlation is significant at the 0.01 level; *means that correlation is significant at the 0.05 level. IS, work-related information sharing of supervisors; NIS, non-work information sharing of supervisors; RE, relationship energy; LQC, family-like employee–organization relationship; PC, proactive change behavior.

### Homology Deviation Analysis

In this study, Harman single-factor test was used to test the homology deviation between variables. The test results showed that there are five factors with eigenvalue greater than 1, and the cumulative variance interpretation amount is 68.381%. The variance interpretation amount of the first principal component is 29.185%, less than half of the cumulative variation interpretation amount. Thus, the common method deviation is not so serious.

### Hypotheses Test

Since there are multiple mediated variables in this model, their modes of action may be sequential. Therefore, before the hypothesis testing, it is necessary to identify and judge the optimal reference model to avoid meaningless parameter estimation. In this study, the following sis models are constructed by Mplus software, and the optimal reference model is selected according to the corresponding fitting indexes and discrimination criteria.

Model 1: The mediation model of this study. Model 2: The order of model variables is adjusted as relational energy → family-like employee–organization relationship → work-related information sharing of supervisors → the proactive change behavior of employees. Model 3: The order of model variables is adjusted as family-like employee–organization relationship → relationship energy → work-related information sharing of supervisors → the proactive change behavior of employees. Model 4: A parallel mediation model, including work-related information sharing of supervisors, influences the proactive change behavior of employees through relationship energy and the family-like employee–organization relationship, respectively. Model 5: Full path model, that is, the direct path from work-related information sharing of supervisors to relational energy, the direct path from the family-like employee–organization relationship to the proactive change behavior of employees, and the direct path from work-related information sharing of supervisors to the proactive change behavior of employees are added. Model 6: Work-related information sharing of supervisors affects the proactive change behavior of employees only through mediated effect of the family-like employee–organization relationship and relationship energy.

The fitting index of six models is summarized in Table 3. According to Table 3, except Model 1 and Model 5, the other models fail to meet the standard [χ^2^/df of Model 2, Model 3, and Model 4 are all greater than 3, and standardized root mean square residual (SRMR) of Model 6 is higher than 0.06]. Taking the comparison of Model 1 and Model 5, we found that the differences between its indicators are not significant. Since they belong to nested models, the Chi-square changes of the model should also be observed. When the Chi-square changes are significant, the complex model with better fitting results should be selected. When the Chi-square changes are not significant, the model with more concise path should be selected. The results show that the Chi-square of Model 1 has a significant change compared with Model 5 (Δχ^2^ = 3.079, *P* > 0.05), so Model 1 with a simple path should be chosen.

**TABLE 3:** Structural equation model (SEM) contrast test.

Model	χ^2^	df	χ^2^/df	CFI	TLI	RMSEA	SRMR
Model 1	169.882	83	2.047	0.982	0.977	0.05	0.05
Model 2	262.154	83	3.158	0.963	0.953	0.072	0.09
Model 3	269.28	83	3.244	0.961	0.951	0.073	0.095
Model 4	4896.721	105	46.635	0.985	0.982	0.045	0.043
Model 5	166.803	82	2.034	0.982	0.977	0.05	0.047
Model 6	190.963	85	2.247	0.978	0.973	0.054	0.085

*CFI, comparative fit index; RMSEA, root mean square error of approximation; SD, standard deviation; SRMR, standardized root mean square residual; TLI, Tucker–Lewis index.*

In conclusion, Model 1 has the best fitting effect. Comparison results of Models 1–6 are shown in Table 3.

#### Mediation Effect Test

**FIGURE 2:**
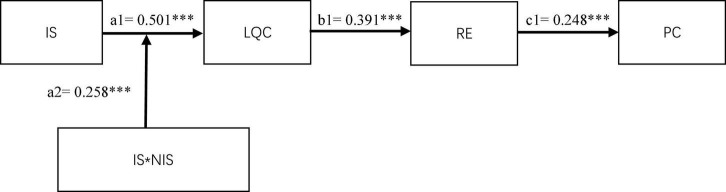
Path coefficient diagram of moderated serial mediation model. It is standard coefficient that show in graph. IS, work-related information sharing of supervisors; NIS, non-work information sharing of supervisors; RE, relationship energy; LQC, family-like employee–organization relationship; PC, proactive change behavior. ^***^Means that the correlation is significant at the 0.001 level; ^**^means that the correlation is significant at the 0.01 level; *means that the correlation is significant at the 0.05 level. In order to keep the graph concise and clear, the path coefficient of the control variable was not drawn into the graphical model.

Having determined the optimal benchmark model, this article used the structural equation model (SEM) and Bootstrap method to test the mediation model. We controlled the control variables. The running results of the benchmark mediation model are shown in the Figure 2, and the Bootstrap test results are shown in the table. The analysis is as follows:

(1) Work-related information sharing of supervisors positively affects the family-like employee–organization relationship (β = 0.501, *P* < 0.001). Hypothesis 1 is verified.
(2) Family-like employee–organization relationship has a positive effect on relationship energy (β = 0.391, *P* < 0.001). Family-like employee–organization relationship has a significant mediation effect on the relationship energy between work-related information sharing of supervisors and relationship energy (β = 0.143, *P* < 0.001). A total of 95% confidence interval is [0.079, 0.225], excluding 0; Hypothesis 2 and Hypothesis 3 are verified.
(3) Relationship energy has a positive impact on the proactive change behavior of employees (β = 0.248, *P* < 0.001). A total of 95% confidence interval is [0.125, 0.375], excluding 0, and Hypothesis 4 is verified.
(4) The serial mediation effect of family-like employee–organization relationship and relationship energy on work-related information sharing of supervisors and the proactive change behavior is significant (β = 0.035, *P* < 0.05). A total of 95% confidence interval is [0.014, 0.072], excluding 0, and Hypothesis 5 is verified.

#### Moderation Effect Test

In this study, the latent moderating structural equation method proposed by Fang and Wen (2018) and the moderated serial mediation model algorithm proposed by Stride et al. (2016) are adopted to test the moderation effect of non-work information sharing of supervisors. The moderated mediation effect analysis based on the latent moderating structural equation method needs to take the acceptability of the SEM as premise. Determining whether the model is acceptable or not requires the following three steps:

In the first step, fitting models are commonly used to determine whether the fitting conditions of the benchmark SEM without interaction terms are acceptable. In this study, *P* < 0.001 for the benchmark model (χ^2^ = 169.882, df = 83, χ^2^/df = 2.047, comparative fit index = 0.982, Tucker–Lewis index = 0.977, root mean square error of approximation = 0.05, and SRMR = 0.05), indicating that the model fitting index is acceptable.

In the second step, we judged whether the fitting condition of the mediation SEM containing interaction terms is better than that of the benchmark model. Sardeshmukh and Vandenberg (2017) believe that the Akaike information criterion (AIC) value can reflect the information loss of the model. And the larger the AIC value is, the more information the model loses. Therefore, judging by the AIC value, if the AIC value becomes smaller or remains the same, it indicates that the model is improved or at least not deteriorated. In this study, the AIC value of the benchmark SEM is 11,924.21, and the AIC value of the serial mediating SEM with latent moderating item is 11,911.459, which is 12.751 less than that of the former, indicating that the SEM with latent moderating item is relatively better.

**FIGURE 3:**
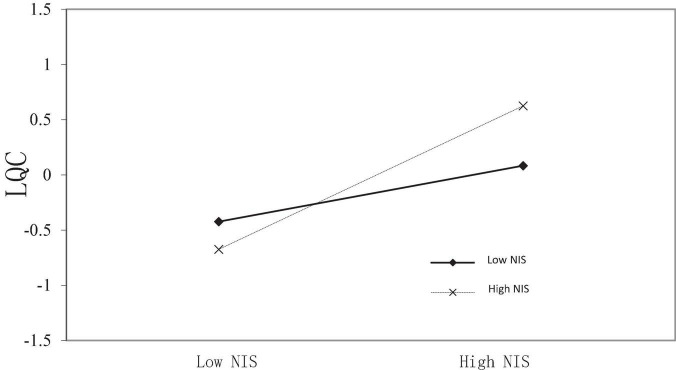
Moderating effect of non-work information sharing of supervisors on the relationship between work-related information sharing of supervisors and family-like employee–organization relationship.

According to the above two steps, it can be seen that the moderated serial mediation SEM in this study is acceptable. Therefore, LMS and Bootstrap are used to analyze the moderated mediating effect, and the results are shown in Figure 3.

The interaction term coefficient of work-related information sharing of supervisors and their non-work information sharing is significant (β = 0.501, *P* < 0.001), which indicates that non-work information sharing of supervisors positively moderates the relationship between their work-related information sharing and family-like employee–organization relationship. That means the more of non-work information sharing of supervisors leads to the greater positive effect of their work-related information sharing on the family-like employee–organization relationship and *vice versa*.

According to the study of Fang and Wen (2018), the moderating serial mediating model can be verified by using the coefficient product rule proposed by Hayes (2013). In this study, we combined the Bootstrap analysis method and LMS, obtaining bias-corrected Bootstrap confidence intervals of some parameters (see Table 4).

**TABLE 4:** Moderated serial mediation effect analysis.

Moderated variable	Path: IS → LQC → RE → TC				
	Effect size	SE	Est./SE	95% confidence interval	
				Lower limit	Upper limit
Coefficient product	0.026	0.011	2.374	0.007	0.051
Non-work information of low supervisors	0.105	0.04	2.613	0.043	0.253
Non-work information of high supervisors	0.165	0.065	2.54	0.055	0.423
Difference	0.061	0.025	2.374	0.017	0.423

As can be seen from Table 4, the effect size of coefficient product is 0.026, and the 95% confidence interval is [0.007, 0.051], which does not include 0. Therefore, according to the coefficient product rule, the moderating serial mediation effect in this study is significant. When the degree of non-work information sharing of supervisors is low, the serial mediating effect size of their work-related information sharing on the proactive change behavior of employees through the family-like employee–organization relationship and relationship energy is 0.105, with confidence interval of [0.043, 0.253], excluding 0. When the degree of non-work information sharing of supervisors is high, the serial mediating effect size of their work-related information sharing on the proactive change behavior of employees through the family-like employee–organization relationship and relationship energy is 0.165, with confidence interval of [0.055, 0.423], excluding 0. The difference value represents the difference of indirect effect size of serial mediated path between high and low non-work information sharing of supervisors. The difference value is 0.061, and 95% Bootstrap confidence interval is [0.017, 0.423]. In particular, supervisors share more non-work information, so the serial mediating effect of their work-related information sharing on the proactive change behavior of employees through the family-like employee–organization relationship and relationship energy has been significantly enhanced.

According to the results of the confidence interval test, non-work information sharing of supervisors can significantly moderate the serial mediating effect of their work-related information sharing on the proactive change behavior of employees through the family-like employee–organization relationship and relationship energy. Thus, Hypothesis 6 is verified.

## Research Conclusion and Discussion

### Research Conclusion

Based on the self-disclosure theory, this study constructed moderated serial mediated model of work-related information sharing of supervisors affecting the proactive change behavior of employees and discussed that under different degrees of non-work information sharing of supervisors, their work-related information sharing affect the proactive change behavior of employees through the family-like employee–organization relationship and relationship energy. The results show that work-related information sharing of supervisors positively affects the family-like employee–organization relationship; family-like employee–organization relationship has a mediation effect on the relationship between work-related information sharing of supervisors and relationship energy, family-like employee–organization relationship and relationship energy have a serial mediation effect on the relationship between work-related information sharing of supervisors and the proactive change behavior of employees, and non-work information sharing of supervisors positively moderates the relationship between work-related information sharing of supervisors and family-like employee–organization relationship. Furthermore, it is verified that non-work information sharing of supervisors has a positive moderating effect on serial mediating effect of their work-related information sharing – family-like employee–organization relationship and relationship energy – and the proactive change behavior of employees.

### Theoretical Significance and Practical Implications

First, this study reveals the mechanism between work-related information sharing of supervisors and the proactive change behavior of employees from the perspective of the self-disclosure theory. This study focuses on the actual empowering leadership behavior of work-related information sharing of supervisors, verifying that it is an important situational factor that affects the proactive change behavior of employees. Therefore, supervisors need to realize the importance of work-related information sharing so as to improve the awareness of the work content of employees and their ability to take risks. Enterprises also need to hold targeted theoretical courses and practical guidance of information sharing behavior, which helps supervisors establish a correct concept of information sharing through a series of leadership training courses. In addition, ordinary employees working in the front line have a better understanding of the problems existing in the daily operation of the enterprise and are an important resource for the enterprise to quickly respond to market changes (Rasool et al., 2021). The proactive change behavior spontaneously generated by employees is very important to the optimization of organizational processes and the improvement of efficiency. Therefore, enterprises should pay attention to and encourage employees to take the proactive change behavior.

Second, this study reveals the serial mediating effect of family-like employee–organization relationship and relationship energy on the relationship between work-related information sharing of supervisors and the proactive change behavior of employees. In response to the appeal of Owens et al. (2016), the relationship energy originated in the West is applied to the background of Chinese collectivism and relationship culture, broadening its application scenarios. In contrast, the Western social exchange theory cannot fully explain the Chinese characteristic employee–organization relationship at present. Based on the viewpoint of Zhu et al. (2015), this study introduces family-like employee–organization relationship into the model to illustrate its important effect on the proactive change behavior of employees in a high uncertain work environment. Research shows that work-related information sharing of supervisors can make employees feel the trust and support of the organization and is conducive to promoting the formation of family-like employee–organization relationship. This positive relationship can improve the psychological function of employees in work and make them more willing to implement the proactive change behavior in the organization. Enterprises should pay attention to the construction of family-like culture by providing team-building activities every day and the formulation of practical training programs to cultivate the family-like employee–organization relationship between employees and the organization.

Finally, this study finds that non-work information sharing of supervisors positively moderates the relationship between work-related information sharing of supervisors and the proactive change behavior of employees through the family-like employee–organization relationship and relationship energy. This research conclusion responds to the call of Nifadkar et al. (2019) and introduces non-work information sharing of supervisors into the research, which has made an important contribution to the further enrichment of his research. The research shows that when the supervisor has a high degree of non-work information sharing, it is easier for employees to integrate into the collective in such an atmosphere. When the supervisor shares work-related information, employees will think that the supervisor really attaches importance to their future development and is more likely to have a family-like employee–organization relationship, believe that supervisors and the organization will provide sufficient support and have high relationship energy, and prefer to implement the proactive change behavior in the organization. Therefore, supervisors should pay attention to the important role of non-work information sharing in the workplace, shorten the psychological distance with employees, and create a harmonious organizational atmosphere.

### Limitations and Future Research Directions

Due to the lack of research experience and resource constraints, this study is still insufficient. First, most of the questionnaires used in this study are developed based on foreign national conditions, which cannot guarantee whether they are suitable for the Chinese cultural situation. Therefore, in the future research, we can further develop a questionnaire suitable for local cultural situation of China. Second, in terms of data collection, the measurement of core variables in this study adopts employee self-assessment and is carried out in the same time period, so it is difficult to further determine the causal relationship of the model. In future research, time series design and other evaluation will be used to collect data, so as to reduce the deviation of common methods.

## Data Availability Statement

The raw data supporting the conclusions of this article will be made available by the authors, without undue reservation.

## Ethics Statement

Ethical review and approval was not required for the study on human participants in accordance with the local legislation and institutional requirements. Written informed consent for participation was not required for this study in accordance with the national legislation and the institutional requirements.

## Author Contributions

X-XZ and CL: conceptualization, writing – original draft, writing – review and editing, methodology, and investigation. X-LW: conceptualization, writing – review and editing, supervision, formal analysis, and methodology. J-NL and SX: conceptualization, writing – review and editing, investigation, and validation. All authors reviewed and approved this article for publication.

## Conflict of Interest

The authors declare that the research was conducted in the absence of any commercial or financial relationships that could be construed as a potential conflict of interest.

## Publisher’s Note

All claims expressed in this article are solely those of the authors and do not necessarily represent those of their affiliated organizations, or those of the publisher, the editors and the reviewers. Any product that may be evaluated in this article, or claim that may be made by its manufacturer, is not guaranteed or endorsed by the publisher.
